# Optogenetic stimulation in the medial prefrontal cortex modulates stimulus valence from rewarding and aversive to neutral states

**DOI:** 10.3389/fpsyt.2023.1119803

**Published:** 2023-04-11

**Authors:** Ying Hao Yu, Arthur C. Tsai, Chen Yin Ou, Cai-N Cheng, Fang Chih Chang, Bai Chuang Shyu, Andrew Chih Wei Huang

**Affiliations:** ^1^Department of Psychology, Fo Guang University, Yilan, Taiwan; ^2^Department of Biotechnology and Animal Science, National Ilan University, Yilan, Taiwan; ^3^Institute of Statistical Science, Academia Sinica, Taipei, Taiwan; ^4^Department of Life Sciences, National Central University, Taoyuan, Taiwan; ^5^Institute of Biomedical Sciences, Academia Sinica, Taipei, Taiwan

**Keywords:** morphine, reward, aversion, cingulate cortex, prelimbic cortex, infralimbic cortex, optogenetics, stimulus valence

## Abstract

**Introduction:**

Understanding the modulations of the medial prefrontal cortex (mPFC) in the valence of the stimulus from rewarding and aversive status to neutral status is crucial for the development of novel treatments for drug addiction. This study addressed this issue and examined whether optogenetic ChR2 photostimulation in the cingulate, prelimbic, and infralimbic cortices of the mPFC regulated the valence of saccharin solution consumption from the rewarding property, the aversive property induced by morphine’s conditioning, and the neutral states *via* saccharin extinction processes after morphine’s conditioning.

**Methods:**

All rats received virus infection, buried optical fiber, optical stimulation, water deprivation, and saccharin solution consumption phases. In Experiment 1, rats were given ChR2 virus infection into the cingulate cortex (Cg1), prelimbic cortex (PrL), and infralimbic cortex (IL) to influence the rewarding saccharin solution consumption under photostimulation. In Experiment 2, rats were given ChR2 or EYFP virus infection into the Cg1, PrL, and IL to alter the saccharin solution consumption in the morphine-induced aversively conditioned taste aversion (CTA) and the saccharin solution consumption in the neutral state following the extinction process under photostimulation. Later, the immunohistochemical staining with c-Fos protein was performed for the Cg1, IL, PrL, nucleus accumbens core, nucleus accumbens shell, central amygdala, basolateral amygdala, ventral tegmental area, and dentate gyrus.

**Results:**

The results showed that optogenetic PrL stimulation decreased the rewarding valence of saccharin solution consumption and increased the morphine-induced, aversive valence of saccharin solution consumption. PrL stimulation decreased the neutral valence of saccharin solution consumption *via* the extinction process. Cg1 optogenetic stimulation increased the rewarding valence of saccharin solution consumption and the aversive valence of saccharin solution consumption induced by morphine in conditioning. Optogenetic IL stimulation increased the aversive valence of saccharin solution consumption induced by morphine *via* conditioning.

**Conclusion:**

Altogether, optogenetic stimulation in the subareas of the mPFC modulated the reward, aversion, and neutral valences of the stimulus and altered neuronal activity in the mPFC, amygdala, nucleus accumbens, and hippocampus. Notably, the change of valence was temporary alternation during light-on related to the light-off periods. However, the findings may provide insights in the development of novel treatments for addictive symptoms.

## 1. Introduction

The valence of stimuli, such as reward and aversion, is crucial for the survival of animals (1–3). Based on environmental stimuli, animals enact behaviors to make approaches or avoidances according to rewarding or aversive valence (1). Neutral stimulus is contingent on the rewarding or aversive valence of the stimulus. The valence of the neutral stimulus can be changed from neutral to rewarding/aversive valence and is formed by conditioned learning (2). The valence of rewarding/aversive conditioned learning is extinguished and becomes the neutral stimulus by the process of extinction (4). Therefore, valence changes of stimuli are essential for learning and memory (5). Previous studies on stimulus valence changes have provided some implications for the amelioration of learning and memory disorders, including drug addiction, dependence, or posttraumatic stress disorders (5).

Various studies have shown that some specific brain areas mediate the valence of the stimulus reaction (6–10). For example, the mesolimbic dopamine system, which projects from the ventral tegmental area (VTA) to the nucleus accumbens (NAc), has been shown to govern reward and reinforcement processing, with the valence of the reward induced by drug addiction (6–10). Moreover, the reward valence of the abused drug was shown to drive addictive individuals to more approaching and compulsive behaviors, such as drug relapse, craving, and desire (11, 12). Until now, no research has examined how to switch the valence of stimuli from rewarding and/or aversive to neutral, or offered a novel treatment for ameliorating addictive symptoms; as such, this study examined this issue.

The medial prefrontal cortex (mPFC) plays multiple roles in the regulation of a variety of brain functions, such as emotional regulation (13), working memory (14), stress response (15), stimulus discrimination (16), and integration of stimulus valence and action (17). Concerning the integration of stimulus valence, the mPFC receives the input messages and provides the stimulus valence and context to regulate addiction-associated behaviors (18). For example, the mPFC meticulously arranges distinct, efferent information from the aversive valence processing in the brain and responds to relevant actions (16). The mPFC projection to the amygdala circuits mediates aversive valence during the abstinence phase (18). The prelimbic cortex (PrL) and infralimbic cortex (IL) of the mPFC also modulate the various valences of the stimulus (17, 19). In an aversive valence behavioral task, the inhibition of PrL was shown to disrupt active but not inhibitory avoidance behaviors; in contrast, IL inactivation interfered with both active and inhibitory avoidance behaviors (19). In a cued rewarding go/no-go task, PrL and IL inactivation was shown to dampen inhibitory reward-seeking behaviors but not active reward-seeking behaviors (19). Another study demonstrated that activation of CB1 transmission changed the morphine reward valence to aversive; inhibition of CB1 transmission enhanced the rewarding valence and threshold in morphine-induced conditioned place preference (CPP) conditioning (20). The activation or inhibition of CB1 transmission within the PrL bidirectionally modulates the rewarding and aversive valences of opiates (20). Therefore, mPFC subareas modulate different valences or switch valences to reward and aversion.

Recently, considerable evidence has indicated that mPFC subareas (e.g., Cg1, IL, and PrL), the basolateral amygdala (BLA), NAc, and the CA3 and dentate gyrus (DG) of the hippocampus exhibit increased expression of c-Fos (i.e., neuronal activity) and p-ERK (i.e., neuronal plasticity) under the aversive valence that the saccharin solution was conditioned with morphine administrations (21). This suggests that these specific brain areas are involved in the aversive valence for the saccharin solution suppression by morphine conditioning (21). Moreover, the PrL and IL of the mPFC exhibited more c-Fos expression in the neutral valence of the saccharin solution consumption in the extinction process after aversively conditioning. Therefore, the PrL and IL seem to contribute to the neutral valence of the aversively saccharin solution suppression in the extinction process (21). Therefore, the mPFC, BLA, NAc, and hippocampus were involved in the aversive and neutral valence in morphine-induced conditioning and extinction.

Lesion of the VTA (but not the periaqueductal gray matter; PAG) with NMDA injection was also shown to impair morphine-induced reward in CPP and aversion in conditioned taste aversion (CTA). This indicates that the VTA, but not the PAG, is involved in the reward and aversion responses induced by morphine administration (22). Therefore, the Cg1, PrL, IL, BLA, CeA, NAc, VTA, and the hippocampal CA3 and DG were selected to assess for aversive saccharin solution consumption induced by morphine in conditioning and the extinction process.

The present study included two experiments. In Experiment 1, ChR2 photostimulation was applied in the Cg1, PrL, and IL. This experiment used the animal model of the saccharin solution consumption task and examined how optogenetic ChR2 photostimulation affected the rewarding valence of stimuli. In Experiment 2, ChR2 or EYFP virus was microinjected into the Cg1, PrL, and IL, and then ChR2 or EYFP photostimulation was given into the Cg1, PrL, and IL. This experiment used the animal model of the morphine-induced aversively CTA conditioning and examined whether photostimulation altered the saccharin solution consumption to test the aversive valence of stimuli. Experiment 2 also used the CTA extinction process and tested whether photostimulation affected the saccharin solution consumption for assessing the neutral valence of stimuli.

Altogether, this study used the CaMKII promoter and excitatory ChR2 virus to infect and activate glutamatergic neurons in the Cg1, PrL, or IL, and examined the following issues: first, we examined whether optogenetic Cg1, PrL, and IL stimulation altered the rewarding valence of saccharin solution consumption. Second, we examined whether optogenetic stimulation affected the aversive property of saccharin solution induced by morphine during conditioning as following the previous animal model (23). Third, we examined whether optogenetic Cg1, PrL, and IL stimulation altered the neutral valence of saccharin solution consumption in morphine extinction. Finally, we examined whether the mPFC (e.g., Cg1, PrL, IL), NAc (e.g., NAc core and NAc shell), amygdala (e.g., BLA and CeA), VTA, and hippocampus (e.g., DG) contributed to morphine-induced aversive or neutral valences for saccharine solution consumption during conditioning and extinction following Cg1, PrL, or IL photostimulation.

## 2. Materials and methods

### 2.1. Animals

A total of 122 male Sprague Dawley rats were purchased from BioLasco Taiwan Co., Ltd. At the beginning of the experiment, the weight of each rat was 250–350 g. The rats were housed in a plastic home cage (47 cm long × 26 cm wide × 21 cm high) with hardwood laboratory bedding (Beta Chip). Rats were pair-housed in the home cage. The colony room was maintained at a constant temperature (approximately 23 ± 2°C) with a 12-h light/dark cycle (lights on 6:00–18:00). Except for water deprivation, water and food were allowed freely. All experiments were performed in compliance with the American Psychological Association’s ethical standards for the treatment of the animals. Approval of animal experiments was granted by the Fo Guang University Institutional Animal Care and Use Committee. Every effort was made to minimize animal suffering and the number of animals used. The present study included two experiments. In Experiment 1, 25 rats were microinjected with the ChR2 virus into the Cg1, PrL, and IL, and they were assigned to the Cg1 (*n* = 8), PrL (*n* = 8), and IL (*n* = 9) groups for rewarding saccharin solution consumption (Table 1). Experiment 2 included 97 rats and was divided into conditioning and extinction tests under ChR2 photostimulation for aversive saccharin solution consumption associated with morphine-induced CTA conditioning. In the conditioning test, 51 rats were assigned to the EYFP (*n* = 8) and ChR2 (*n* = 7) groups under photostimulation of the Cg1, EYFP (*n* = 8) and ChR2 (*n* = 10) groups under photostimulation of the PrL, and the EYFP (*n* = 8) and the ChR2 (*n* = 10) groups under photostimulation of the IL. In the extinction tests, 46 rats were assigned to the EYFP (*n* = 7) and ChR2 (*n* = 8) groups under photostimulation of the Cg1, EYFP (*n* = 8) and ChR2 (*n* = 7) groups under poststimulation of the PrL, and EYFP (*n* = 8) and ChR2 (*n* = 8) groups under photostimulation of the IL (Table 1). Note that, 46 rats in extinction that received the procedure of conditioning and extinction; however, 51 rats in conditioning were only subjected to the procedure of conditioning. During these experiments, 12 rats were excluded from our statistical analysis due to incorrect cannula placement (*n* = 9) or lost head caps (*n* = 3), resulting in a final animal number of 122.

**TABLE 1:** Numbers of rats for each group in Experiments 1 and 2.

Experiment 1 (*n* = 25)			
	Cg1 (*n* = 8)		
	PrL (*n* = 8)		
	IL (*n* = 9)		
**Experiment 2 (*n* = 97)**			
Conditioning (*n* = 51)			
		EYFP	ChR2
	Cg1	(*n* = 8)	(*n* = 7)
	PrL	(*n* = 8)	(*n* = 10)
	IL	(*n* = 8)	(*n* = 10)
Extinction (*n* = 46)			
		EYFP	ChR2
	Cg1	(*n* = 7)	(*n* = 8)
	PrL	(*n* = 8)	(*n* = 7)
	IL	(*n* = 8)	(*n* = 8)

### 2.2. Surgery

#### 2.2.1. Virus microinjection and optical fiber implantation

The rats were injected with atropine sulfate (0.1 mg, i.p.) and gentamicin (40 mg/kg, i.p.) 20 min before anesthesia to prevent phlegm and infection, respectively. The rats were then anesthetized with sodium pentobarbital (50 mg/kg, i.p.) and placed in a stereotaxic apparatus. Rats were then microinjected with left infusions of an AAV virus with CaMKII promoter in order to infect neurons [AAV5-CaMKIIa-hChR2(H134R)-EYFP or AAV5-CaMKIIa-EYFP; UNC Vector Core, Chapel Hill, NC]. The target sites of the virus infusions were the Cg1 (bregma AP = +3.24 mm, ML = +0.60, DV = –2.2 mm), PrL (bregma AP = +3.24 mm, ML = +0.60, DV = –3.0 mm), and IL (bregma AP = +3.24 mm, ML = +0.60, DV = –4.8 mm). A total of 0.5 μL of virus was infused over 5 min at a constant rate (0.1 μL/min), and the injector was subsequently kept in place for an additional 10 min to allow for diffusion. After surgery, the virus infection was allowed to develop over 4 weeks. During the infection time, the rats were given food and water *ad libitum*.

At the end of the 4-week virus infection period, all rats were subjected to a surgical procedure to implant an optical fiber into the target brain area and then were allowed to recover for 1 week.

#### 2.2.2. Virus vectors and optical stimulation

AAV5-CaMKIIa-EYFP (titer = 4.1 × 10^12^ vg/mL) and AAV5-CaMKIIa- hChR2(H134R)-EYFP (titer = 4.3 × 10^12^ vg/mL) were purchased from the University of North Carolina viral vector core (Chapel Hill, NC, USA). Optical fibers (0.2 mm in diameter) were inserted into the implanted cannula, and the previous study was referred to for light parameters (24). Laser light was delivered by pulse trains of light with a 473-nm light laser, ∼9 mW/mm^2^, and 15-ms pulses at 20 Hz for 30 s (24). In Experiment 1, all rats were microinjected with AAV5-CaMKIIa-hChR2(H134R)-EYFP in the Cg1, PrL, and IL regions to infect neurons expressing hChR2-EYFP. In Experiment 2, rats in the EYFP and ChR2 groups were microinjected with AAV5-CaMKIIa-EYFP and AAV5-CaMKIIa-hChR2(H134R)-EYFP into the Cg1, PrL, and IL to infect neurons that expressed hChR2-EYFP or EYFP.

### 2.3. Lickometer

The lickometer was comprised of a 25-mL burette with 0.1 mL graduation, a white panel, and a wire-mesh cage. The burette was mounted in front of the wire-mesh cage and linked to the white panel. The consumption volume of the saccharin solution was measured for analysis.

### 2.4. Behavioral procedure

**FIGURE 1:**
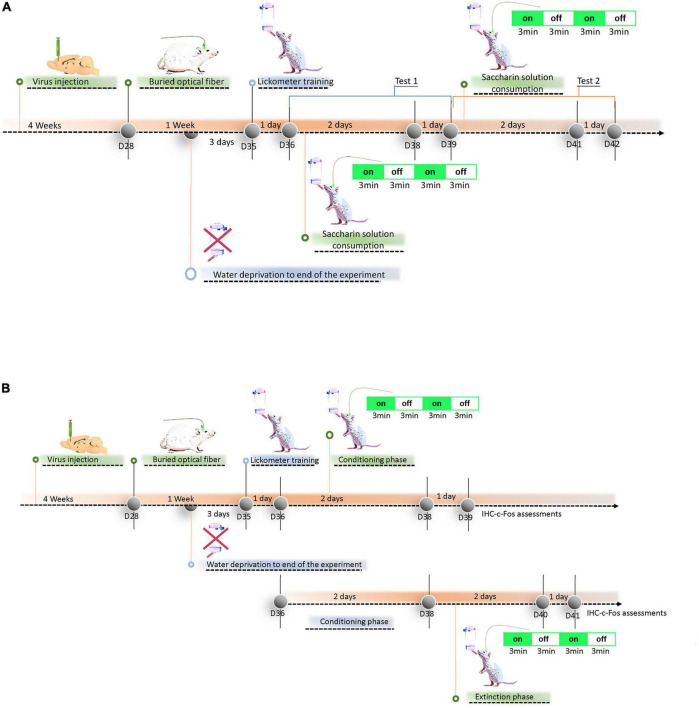
Overview of experimental procedure. **(A)** Experiment 1: The experimental timeline included: virus injection and infection phase for 4 weeks, buried optical fiber and recovery from surgery for 1 week, water deprivation until the end of the experiment, lickometer drinking training for 1 day, 0.1% saccharin solution consumption with 3-min light-on and 3-min light-off treatment over 12 min for 2 days, and drinking 0.1% saccharin solution consumption with the light off over 12 min for 1 day in Test 1. The same cycle and experimental procedure were conducted for Test 2. **(B)** Experiment 2: The experimental timeline included: virus injection and infection phase for 4 weeks, buried optical fiber and recovery from surgery for 1 week, water deprivation to the end of the experiment, lickometer drinking training for 1 day, 0.1% saccharin solution consumption and conditioning with morphine for 3-min light-on and 3-min light-off treatment over 12 min for 2 days, and 0.1% saccharin solution consumption with light off over 12 min for 1 day. After virus injection, buried optical fiber, and lickometer drinking water for 1 day, the other rats received 0.1% saccharin solution conditioned with morphine injections for 2 days in the conditioning phase. Then, the rats were given the 0.1% saccharin solution without morphine injection in the extinction phase. In the extinction phase, 3-min light-on and 3-min light-off treatment was performed over 12 min. The next day, all rats drank 0.1% saccharin solution freely with the light off for 12 min. After behavioral tests, immunohistochemical staining was performed for c-Fos proteins. Note that D28, D35, D36, D38, D39, and D40–42 indicate days 28, 35, 36, 38, and 40–42, respectively.

The present study was composed of two experiments. Experiment 1 is summarized in Figure 1A. At the beginning of the experiment, all rats were given the virus microinjection in the specific left-brain areas (e.g., Cg1, PrL, or IL), and infection was allowed to develop over 4 weeks. Later, all rats were implanted with an optical fiber in the left Cg1, PrL, or IL (Day 28). In the last 3 days (Days 33–35), the water deprivation procedure was conducted until the end of the experiment. During the water deprivation procedure, all rats were given water deprivation for 23.5 h/day. In the evening, the rat was allowed to drink water for 30 min in the home cage. On the next day (Day 36), all rats were trained to drink water freely using the lickometer device for 15 min. For the next 2 days (Days 37–38; i.e., sessions 1–2), rats were allowed to drink the 0.1% saccharin solution for 12 min each day. All rats received a 3-min light-off and a 3-min light-on for sessions 1–2. On the following day (Day 39; i.e., session 3), all rats drank the 0.1% saccharin solution with a 12-min light-off. Sessions 1–3 were served as Test 1 (Days 37–39). Saccharin solution consumption was repeated in the same procedure in Test 2 (Day 40–42; Figure 1A).

Experiment 2 was divided into conditioning and extinction tests under ChR2 photostimulation. In the conditioning test, all rats experienced the same experimental procedure of virus injection and infection development for 4 weeks (Days 1–28), buried optical fiber and recovery for 1 week (Days 29–35), water deprivation for three days (Days 33–35) to the end of the experiment, and licking water training in the lickometer for 1 day (Day 36). For the next 2 days (Days 37–38), the rats drank 0.1% saccharin solution for 12 min, then 20 mg/kg of morphine was intraperitoneally injected for CTA conditioning. During the conditioning phase, ChR2 photostimulation was provided for one cycle with the light on for 3 min and light off for 3 min. All rats received ChR2 photostimulation for a total of two cycles. The next day (Day 39), all rats drank 0.1% saccharin solution for 12 min with light-off treatments. Immunohistochemical staining with c-Fos was performed 120 min after completing behavioral tests. In the extinction test, the rats underwent a similar procedure; however, they underwent the conditioning procedure without ChR2 photostimulation (Days 37–38). The rats were then subjected to the extinction procedure for 2 days (Days 39–40). During the extinction procedure (Days 39–40), the rats were allowed to drink 0.1% saccharin solution without any drug injection for 12 min, and they received ChR2 photostimulation with two cycles of 3 min of light on and 3 min of light off. The next day (Day 41), all rats underwent the same procedure and drank 0.1% saccharin solution with light-off ChR2 photostimulation for 12 min. Later, immunohistochemical staining with c-Fos was performed (Figure 1B).

### 2.5. Immunohistochemical staining

After sacrificing with overdose of sodium pentobarbital and ensuring that the rats were completely unresponsive, the rats were perfused with a 0.1 M sodium phosphate-buffered saline (PBS) buffer followed by 4% paraformaldehyde in a 0.1 MPBS buffer. The brain tissues were then dissected, post-fixed with 4% paraformaldehyde solution, and transferred to 30% sucrose for cryoprotection until the brain sank to the bottom of the solution. The brain tissues were cut into 40-μm coronal sections on a freezing microtome (25). All sections of the brain tissue received c-Fos immunostaining. Furthermore, free-floating brain sections were washed once for 15 min in 0.1 M PBS, permeabilized in 3% H_2_O_2_ for 1 h, washed three times in 2% PBST for 20 min, and soaked in 3% normal goat serum and 1% bovine serum albumin for 1 h. All sections were washed for 15 min in PBST solution. For c-Fos labeling, the sections were incubated at 4°C overnight with rabbit anti-Fos antibody (Millipore, ABE457, 1:1000). Sections were washed twice with PBST for 15 min and incubated with a biotinylated goat anti-rabbit secondary antibody (Vector Lab BA-1000, 1:500) for 1 h. The sections were washed for 10 min with PBS, and the bound secondary antibody was amplified using the ABC kit (Vector Lab ABC Kit, PK-6100). The sections were incubated by a peroxidase staining (2 mg/mL DAB + 3% H_2_O_2_) following the procedure of the ABC kit to stain c-Fos proteins.

Expression of the c-Fos protein was quantified for the whole brain using ImageJ software (26). Counting was performed visually at 20 × magnification for each section of brain tissue. Every third section of each brain slice was selected for counting. Neuron counts for all sections within each brain subarea were averaged for each group. Furthermore, the c-Fos density positive neurons were analyzed using the formula: c-Fos numbers/the slice areas (0.525 mm x 0.833 mm ≒ 0.43725 mm^2^).

### 2.6. Drugs

Morphine hydrochloride was obtained from the Pharmaceutical Plant of the Food and Drug Administration, Ministry of Health and Welfare, Executive Yuan (Taipei, Taiwan). The other chemical compounds were purchased from Sigma-Aldrich (St. Louis, MO, USA). A 20 mg/kg dose of morphine hydrochloride was used in all experiments. Morphine hydrochloride was dissolved in normal saline to a concentration of 20 mg/mL. Sodium chloride was dissolved in distilled water to produce a 0.15 M normal saline solution. Sodium saccharin was prepared in distilled water to produce a 0.1% saccharin solution. Normal saline and morphine were intraperitoneally injected. The injection volume of normal saline and morphine was 1 mL/kg.

### 2.7. Statistical analysis

Each session indicates each day. The mean (±SEM) total intake volume of saccharin solution was merged the intake volume of saccharin solution for twice light-off and light-on treatments for sessions 1–2 in the Cg1, PrL, and IL. In Experiment 1, the mean (±SEM) total intake volume of saccharin solution was analyzed by dependent *t*-test of light-off and light-on treatments in the Cg1, PrL, and IL for each session in Tests 1–2. Furthermore, it was analyzed by dependent *t*-test for the mean (±SEM) intake volume of saccharin solution between light-off and light-on on sessions 1–2 and between the first light-off and the later light-off on session 3 in Tests 1–2. In Experiment 2, the mean (±SEM) total intake volume of saccharin solution was analyzed by a two-way repeated-measures mixed ANOVA for light-off and light-on treatments in the EYFP and ChR2 groups in the Cg1, PrL, and IL during conditioning and extinction phases.

In some cases, to identify whether the intake volume of saccharin solution was significantly different between light-off or light-on treatments, a one-way ANOVA was performed to evaluate the mean (±SEM) intake volume of saccharin solution for light-off and light-on sessions between the EYFP and ChR2 groups. Additionally, the mean (±SEM) intake volume of saccharin solution was analyzed by one-way ANOVA in light-on 1, light-off 1, light-on 2, light-off 2, and twice light-off 3 between the EYFP and ChR2 groups in the Cg1, PrL, and IL during conditioning and extinction phases. *p* < 0.05 was considered statistically significant.

If the behavior testing results were significantly different, the c-Fos expression data were analyzed by one-way ANOVA for the Cg1, PrL, IL, NAc core, NAc shell, BLA, CeA, DG, PC, and VTA.

## 3. Results

### 3.1. Experiment 1: Rewarding saccharin solution consumption

To test whether the Cg1, PrL, and IL of the mPFC modulated the rewarding valence in the saccharin solution consumption, Experiment 1 used optogenetic ChR2 photostimulation to excite the Cg1, PrL, and IL and test the rewarding saccharin solution consumption.

In Experiment 1, all rats were randomly assigned into groups and were microinjected with the ChR2 excitatory virus in the Cg1, PrL, and IL regions of the brain. Then, the rats were given the 0.1% saccharin solution consumption for 12 min per day over three days in Test 1. Later, all rats were allowed to drink the 0.1% saccharin solution for 12 min per day for another three days in Test 2. Virus infection in the target brain areas was verified next (Supplementary Figure 1A). To verify infection in the specific brain areas, the rat brain atlas was used to visualize the ChR2 microinjections in the Cg1, PrL, and IL. These infected the subjects with glutamatergic neurons expressing ChR2-EYFP proteins under the CaMKIIa promoter in green, label c-Fos protein expression in red, and merge fluorescence ChR2 and c-Fos expression. Schematic depictions show optical cannula placement in the Cg1 (blue dots), PrL (orange dots), and IL (green dots). The blue, orange, and green dots depict the ventral point of the cannula tract. Some rats were excluded due to cannula placement outside the target areas (Supplementary Figure 1B).

**FIGURE 2:**
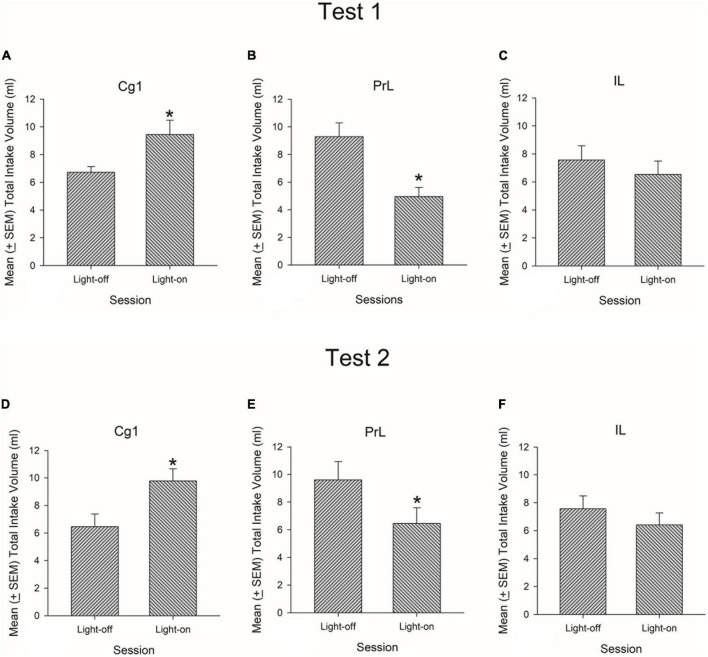
Mean (±SEM) total intake volume of saccharin solution in rats with exposure to 3-min light-on and 3-min light-off optical stimulation twice over each 12 min session in the Cg1, PrL, and IL in Tests 1 **(A–C)** and 2 **(D–F)**. *p < 0.05 indicates a significant difference between the light-on and light-off sessions.

Cg1 photostimulation significantly enhanced the intake volume of 0.1% saccharin solution during light-on related to light-off periods during the session [*t*(7) = –2.92, *p* < 0.05; Figure 2A], while PrL photostimulation significantly decreased the intake volume of 0.1% saccharin solution on light-on compared to the light-off period during the session [*t*(7) = 5.32, *p* < 0.05; Figure 2B]. However, in Test 1, IL photostimulation did not alter the intake volume of 0.1% saccharin solution between light-off and light-on sessions [*t*(8) = 0.98, *p* > 0.05; Figure 2C]. The results of Test 2 were similar to those of Test 1: Cg1 photostimulation significantly increased the intake volume of 0.1% saccharin solution on light-on related to light-off during the session [*t*(7) = –3.69, *p* < 0.05; Figure 2D], PrL photostimulation significantly decreased the intake volume on light-on related the light-off period on this session [*t*(7) = 3.42, *p* < 0.05; Figure 2E], and IL photostimulation did not influence the intake volume between light-off and light-on sessions [*t*(8) = 1.32, *p* > 0.05; Figure 2F].

**FIGURE 3:**
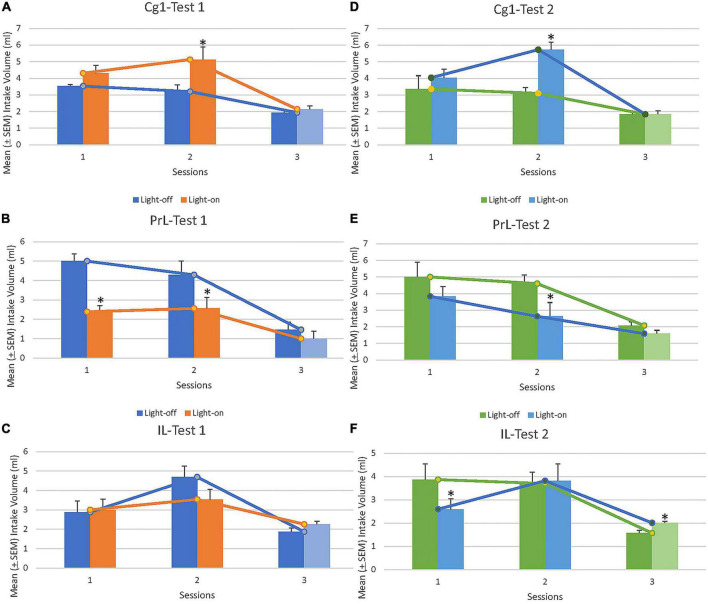
Mean (±SEM) intake volume of saccharin solution in rats in light-on and light-off conditions following optical stimulation of Cg1, PrL, and IL for Sessions 1 and 2, Tests 1 **(A–C)** and 2 **(D–F)**. In Session 3, the rats drank saccharin solution with the lights off for 12 min. *p < 0.05 indicates a significant difference between the light-on and light-off sessions. Note that, light and dark blue or green vertical bars indicated light-off treatments in Test 1 and Test 2. In summary, the Cg1 increased the rewarding saccharin solution consumption; however, the PrL decreased the rewarding saccharin solution consumption. The IL did not affect the rewarding valence of the saccharin solution consumption.

Furthermore, ChR2 photostimulation of the Cg1, PrL, and IL was evaluated over Sessions 1 and 2 (i.e., 3-min light-off and 3-min light-on treatments) and all light-off treatments for Session 3 to evaluate whether the 0.1% saccharin solution consumption changed. In Test 1, light-on photostimulation of Cg1 increased the intake volume of 0.1% saccharin solution in Session 2 [*t*(7) = –2.91, *p* < 0.05] but not in Session 1 [*t*(7) = –1.64, *p* > 0.05] or Session 3 [*t*(7) = –0.70, *p* > 0.05; Figure 3A] when compared to the light-off period. The results of Test 2 were consistent with those of Test 1: Cg1 photostimulation increased the intake volume of 0.1% saccharin solution in Session 2 [*t*(7) = –5.50, *p* < 0.05] but not in Session 1 [*t*(7) = –0.90, *p* > 0.05] or Session 3 [*t* (7) = 0.00, *p* > 0.05; Figure 3D] on light-on related to the light-off period.

In Test 1, light-on photostimulation of PrL decreased the intake volume of 0.1% saccharin solution in Session 1 [*t*(7) = 6.67, *p* < 0.05] and Session 2 [*t*(7) = 2.37, *p* = 0.05] related to the light-off period; however, no significant change in intake volume was observed in Session 3 [*t*(7) = 1.52, *p* > 0.05; Figure 3B] between light-on and light-off periods. In Test 2, light-on photostimulation of PrL significantly decreased the intake volume of 0.1% saccharin solution in Session 2 [*t*(7) = 2.28, *p* = 0.05] compared to the light-off period, but the intake volume of 0.1% saccharin solution did not significantly change between light-off and light-on treatments in Session 1 [*t*(7) = 2.09, *p* > 0.05] or Session 3 [*t*(7) = 1.79, *p* > 0.05; Figure 3E].

In Test 1, light-on photostimulation of IL did not affect the intake volume of 0.1% saccharin solution in Session 1 [*t*(8) = –0.21, *p* > 0.05], Session 2 [*t*(8) = 1.66, *p* > 0.05], or Session 3 [*t*(8) = –1.91, *p* > 0.05; Figure 3C] related to the light-off period. In Test 2, IL photostimulation decreased the intake volume of 0.1% saccharin solution in Session 1 [*t*(8) = 2.84, *p* < 0.05] and, in Session 3, there were significant differences in the intake volume of 0.1% saccharin solution [*t*(8) = –4.59, *p* < 0.05] for two light-off conditions under IL photostimulation; however, no significant difference in intake was observed between light-on and light-off conditions in Session 2 [*t*(8) = –0.16, *p* > 0.05; Figure 3F].

### 3.2. Experiment 2: Aversive saccharin solution suppression induced by morphine in conditioning and extinction

Experiment 2 examined whether the Cg1, PrL, and IL of the mPFC modulated the aversive valence of the saccharin solution suppression induced by morphine in conditioning and the neutral valence of the extinction process in extinction. In Experiment 2, rats were given the 0.1% saccharin solution consumption for 12 min and then intraperitoneally injected with 20 mg/kg morphine to initiate aversive saccharin solution consumption suppression during the conditioning phase. Later, in the extinction phase, rats were subjected to 0.1% saccharin solution consumption without morphine injections to produce the extinction effect. All rats were randomly assigned to control EYFP or ChR2 excitatory virus infection groups. Later in the experiment, optogenetic ChR2 excitatory photostimulation of the Cg1, PrL, and IL of the mPFC was applied to examine whether the treatment affected morphine-induced aversive saccharin solution suppression in cases of conditioning and extinction.

First, viral infection was verified (Supplementary Figure 2). To visualize the target brain areas (i.e., Cg1, PrL, and IL) of ChR2 or EYFP virus infection and c-Fos protein expression after photostimulation, the ChR2 virus infection was marked with green fluorescence, c-Fos expression was marked with red fluorescence, and ChR2 and c-Fos expression in the Cg1, PrL, and IL was merged (Supplementary Figure 2A). Viral infections with c-Fos activation showed that the Cg1 and PrL were similar in a similar state as during the activation of c-Fos neurons; however, the IL revealed a lower activation in c-Fos neurons under ChR2 photostimulation. The verification of viral infection was shown as follows.

The EYFP microinjection group exhibited 30.96% c-Fos expression and the ChR2 group exhibited 43.97% c-Fos expression following Cg1 photostimulation. Following PrL photostimulation, the EYFP group exhibited 40.48% c-Fos expression and the ChR2 group exhibited 43.08% c-Fos expression. IL photostimulation produced 36.76% c-Fos expression in the EYFP group and 54.09% c-Fos expression in the ChR2 group (Supplementary Figure 2B).

During conditioning and extinction tests, the EYFP and ChR2 groups were located using anatomical diagrams (Supplementary Figure 2C). Optical cannula placement is depicted in the EYFP group within the Cg1 (blue dots), PrL (orange dots), and IL (green dots). The cannula placement in the ChR2 group was in the Cg1 (gray dots), PrL (brown dots), and IL (dark green dots). Blue, orange, green, gray, brown, and dark green dots depict the ventral point of the cannula tract (Supplementary Figure 2C). The rats with cannula placement outside the target areas were excluded from the study.

#### 3.2.1. Behavioral data: Optogenetic photostimulation in specific brain areas during light-off and light-on sessions

**FIGURE 4:**
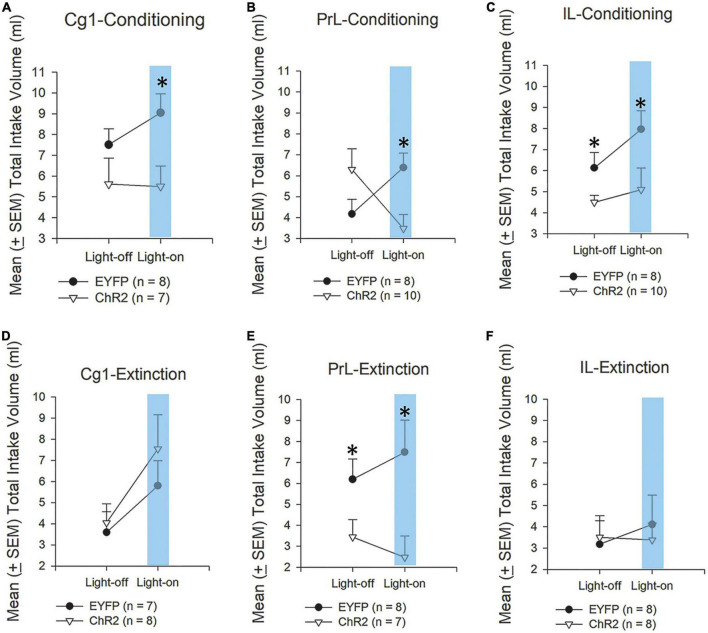
Morphine acts as an aversive unconditioned stimulus (US) in CTA. Mean (±SEM) total intake volume of saccharin solution in rats injected with morphine under optical stimulation of the Cg1, PrL, and IL during conditioning **(A–C)** and extinction **(D–F)**; 3-min light-on and 3-min light-off treatments were conducted over two sessions. In the CTA test, the rats were divided into EYFP (*n* = 8) and ChR2 (*n* = 7) groups. *p < 0.05 indicates the ChR2 group compared with the EYFP group. Note that comparison of means for light-on conditions are therefore made up of 1 measure without CTA and one measure with CTA.

We examined behavioral data indicated to examine whether optogenetic ChR2 excitatory photostimulation in the target brain areas could affect saccharin solution consumption induced by morphine (Figures 4A–C). Following Cg1 photostimulation and conditioning tests, the intake volume of saccharin solution in the ChR2 group was significantly lower than in the EYFP group [*F*_(1, 13)_ = 7.68, *p* < 0.05]. Non-significant differences occurred in session (i.e., light-off and light-on treatments) [*F*_(1, 13)_ = 0.52, *p* > 0.05] and group × session [*F*_(1, 13)_ = 0.71, *p* > 0.05]. Notably, the intake volume of saccharin solution in the light-on sessions was significantly decreased [*F*_(1, 13)_ = 6.99, *p* < 0.05; Figure 4A].

In the PrL photostimulation and conditioning tests, there was a significant interaction between group × session and the intake volume of saccharin solution [*F*_(1, 16)_ = 38.95, *p* < 0.05]. Non-significant differences occurred in group [*F*_(1, 16)_ = 0.38, *p* > 0.05] and in session [*F*_(1, 16)_ = 2.53, *p* > 0.05]. Furthermore, the intake volume of saccharin solution in the ChR2 group was significantly decreased compared with the EYFP group in the light-on session [*F*_(1, 16)_ = 8.98, *p* < 0.05; Figure 4B].

In the IL photostimulation and conditioning tests, the intake volume of saccharin solution was significantly decreased in the ChR2 group compared with the EYFP group [*F*_(1, 16)_ = 7.38, *p* < 0.05] in light-off and light-on sessions. However, non-significant differences occurred in session [*F*_(1, 16)_ = 2.60, *p* > 0.05] and group × session [*F*_(1, 16)_ = 0.67, *p* > 0.05; Figure 4C].

This study also tested whether optogenetic ChR2 photostimulation in the Cg1, PrL, and IL impacted the extinction effect following morphine-induced saccharin solution consumption suppression (Figures 4D–F).

The results showed that under ChR2 photostimulation of Cg1, non-significant differences occurred in group [*F*_(1, 13)_ = 0.47, *p* > 0.05] and group × session [*F*_(1, 13)_ = 0.98, *p* > 0.05]. However, there was a significant difference between light-on and light-off treatments [*F*_(1, 16)_ = 19.69, *p* < 0.05; Figure 4D].

ChR2 photostimulation of PrL in extinction tests showed that the intake volume of saccharin solution in the ChR2 group was significantly decreased compared to in the EYFP group in both the light-off and light-on sessions [*F*_(1, 13)_ = 6.40, *p* < 0.05]. In group × session, there was significant interaction with the intake volume [*F*_(1, 13)_ = 4.64, *p* = 0.05]. However, only a non-significant difference occurred in session [*F*_(1, 13)_ = 0.10, *p* > 0.05; Figure 4E].

In IL photostimulation and extinction tests, non-significant differences occurred in group [*F*_(1, 14)_ = 0.02, *p* > 0.05], session [*F*_(1, 14)_ = 1.21, *p* > 0.05], and group × session [*F*_(1, 14)_ = 2.18, *p* > 0.05; Figure 4F].

**FIGURE 5:**
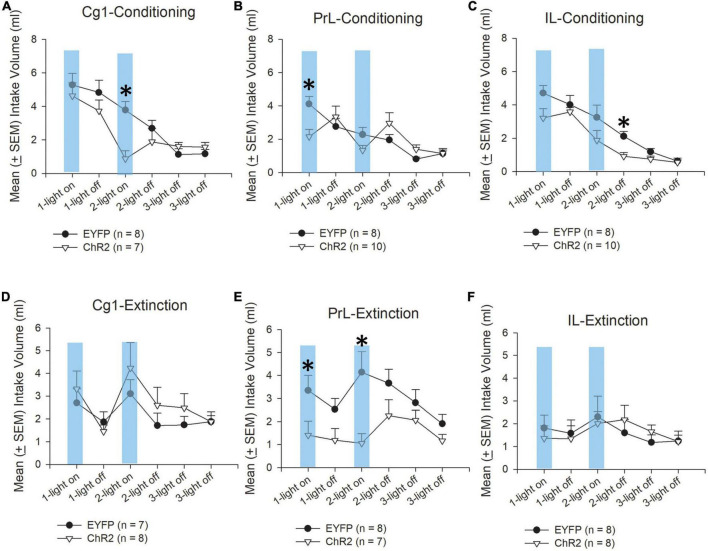
Morphine acts as an aversive US in CTA. Mean (±SEM) intake volume of saccharin solution in rats injected with morphine under optical stimulation of the Cg1, PrL, and IL during conditioning **(A–C)** and extinction **(D–F)**, with 3-min light-on and 3-min light-off treatments occurring over two sessions and 3-min light-off and 3-min light-off treatments occurring in Session 3. In the CTA test, the rats were divided into EYFP (*n* = 8) and ChR2 (*n* = 7) groups. *p < 0.05 indicates the ChR2 group compared with the EYFP group.

Further behavioral analysis under light-on and light-off conditions over three sessions were conducted to measure the issue of whether ChR2 photostimulation in the Cg1, PrL, and IL affected morphine-induced saccharin solution suppression and its extinction (Figure 5). Mean (±SEM) intake volume (mL) was measured over three cycles of light-on and light-off treatment under ChR2 optogenetic photostimulation of the Cg1, PrL, and IL during conditioning.

Regarding conditioning tests, Cg1 photostimulation revealed that the differences in intake volume of saccharin solution were non-significant in group [*F*_(1, 13_) = 3.94, *p* > 0.05]. Significant differences occurred in session [*F*_(5, 65)_ = 16.08, *p* < 0.05] and group × session [*F*_(5, 65)_ = 2.71, *p* < 0.05]. Furthermore, there was a significant decrease in the intake volume of saccharin solution following light-on treatment in Session 2 [*F*_(1, 13)_ = 16.72, *p* < 0.05; Figure 5A].

PrL photostimulation showed that the differences in intake volume of saccharin solution were non-significant in group [*F*_(1, 13)_ = 0.09, *p* > 0.05]. Significant differences occurred in session [*F*_(5, 80)_ = 14.39, *p* < 0.05] and group × session [*F*_(5, 80)_ = 5.54, *p* < 0.05]. Furthermore, there was a significant decrease in the intake volume of saccharin solution following light-on treatment in Session 1 (*p* < 0.05; Figure 5B).

IL photostimulation showed that there were significant differences in group [*F*_(1, 16)_ = 7.77, *p* < 0.05] and in session [*F*_(5, 80)_ = 27.83, *p* < 0.05]. Non-significant differences occurred in group × session [*F*_(5, 80)_ = 1.18, *p* > 0.05]. Particularly, a decrease in the intake volume of saccharin solution following light-off treatment in Session 2 [*F*_(1, 16)_ = 16.47, *p* < 0.05; Figure 5C] in the ChR2 group.

Regarding the extinction tests, following Cg1 photostimulation, there were non-significant difference in saccharin solution intake volume in group [*F*_(1, 13)_ = 0.48, *p* > 0.05] and group × session [*F*_(5, 65)_ = 0.86, *p* > 0.05]. The intake volumes of saccharin solution in the extinction tests were significantly different in session [*F*_(5, 65)_ = 5.91, *p* < 0.05; Figure 5D].

PrL photostimulation in the extinction tests showed that the intake volume of saccharin solution was significantly different in group [*F*_(1, 13)_ = 5.81, *p* < 0.05], session [*F*_(5, 65)_ = 3.33, *p* < 0.05], and group × session [*F*_(5, 65)_ = 2.42, *p* < 0.05]. Moreover, there was a significant decrease in saccharin solution intake following ChR2 photostimulation in the light-on treatment in Session 1 [*F*_(1, 13)_ = 4.63, *p* = 0.05] and light-on treatment in Session 2 [*F*_(1, 13)_ = 8.70, *p* < 0.05; Figure 5E].

IL photostimulation in extinction tests showed that non-significant differences occurred in group [*F*_(1, 14)_ = 0.00, *p* > 0.05], session [*F*_(5, 70)_ = 1.63, *p* > 0.05], and group × session [*F*_(5, 70)_ = 0.06, *p* > 0.05]. No significant differences were observed in the intake volume of saccharin solution in the ChR2 group compared to the EYFP group for all light-on and light-off sessions (Figure 5F).

In summary, the Cg1, PrL, and IL increased the aversive valence of the saccharin solution suppression induced by morphine in conditioning. Only, the PrL decreased the neutral valence of saccharin solution consumption in extinction, and the Cg1 and IL did not affect the neutral valence in extinction.

Altogether, the subareas of the mPFC played different modulating roles in the rewarding valence of the saccharin solution intake: Cg1 had an upregulating role, and PrL had a downregulating role. IL was not involved in the rewarding valence of saccharin solution consumption (Table 2A). Therefore, optogenetic Cg1 stimulation enhanced the rewarding valence of saccharin solution consumption, and optogenetic PrL stimulation decreased it. On the other hand, optogenetic Cg1, PrL, and IL stimulation increased the aversive valence of saccharin solution consumption induced by morphine administration in conditioning (Table 2B). Only optogenetic PrL stimulation decreased the neutral valence of saccharin solution consumption induced by morphine extinction (Table 2C).

**TABLE 2:** Optogenetic ChR2 excitatory photostimulation of the Cg1, PrL, and IL and measure the following conditions.

(A)		
	**Test 1**	**Test 2**
Cg1	↑	↑
PrL	↓	↓
IL	—	—
**(B)**		
	**Light off**	**Light on**
Cg1	—	↑
PrL	—	↑
IL	↑	↑
**(C)**		
	**Light off**	**Light on**
Cg1	—	—
PrL	↓	↓
IL	—	—

(A) Assess the rewarding valence of the saccharin solution consumption in Test 1 and Test 2. (B) Assess aversive saccharin solution suppression induced by morphine in the conditioning phase. (C) Assess aversive saccharin solution suppression induced by morphine in the extinction phase. —, non-significant differences; ↓, decrease; ↑, increase; Cg1, cingulate cortex 1; PrL, prelimbic cortex; IL, infralimbic cortex.

#### 3.2.2. Neural substrates and c-Fos expression

**FIGURE 6:**
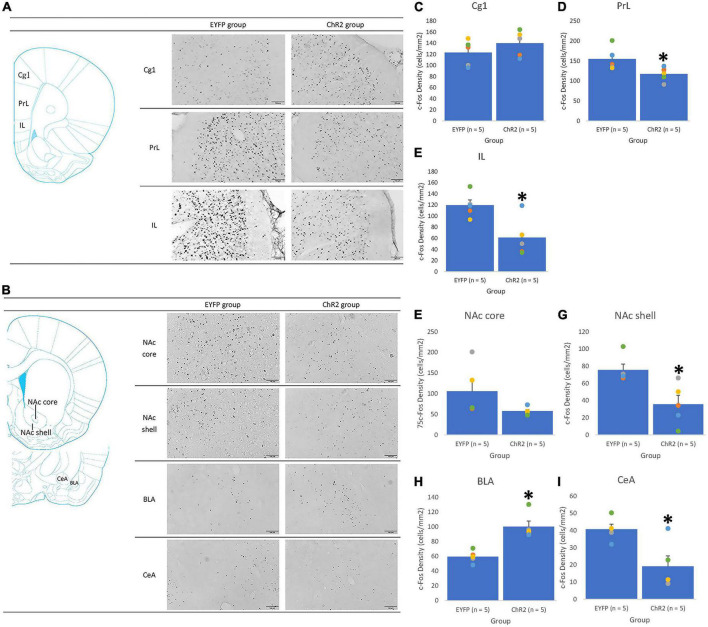
The Cg1 photostimulation was given in conditioning. An illustrated brain atlas showing **(A)** the cingulate cortex 1 (Cg1), prelimbic cortex (PrL), and infralimbic cortex (IL); **(B)** the nucleus accumbens core (NAc core), nucleus accumbens shell (NAc shell), basolateral amygdala (BLA), and central amygdala (CeA). c-Fos density in the **(C)** Cg1, **(D)** PrL, **(E)** IL, **(F)** NAc core, **(G)** NAc shell, **(H)** BLA, and **(I)** CeA in EYFP (*n* = 5) and ChR2 (*n* = 5) groups after Cg1 optical stimulation and morphine-induced CTA in conditioning tests. *p < 0.05 compared with the EYFP group.

**FIGURE 7:**
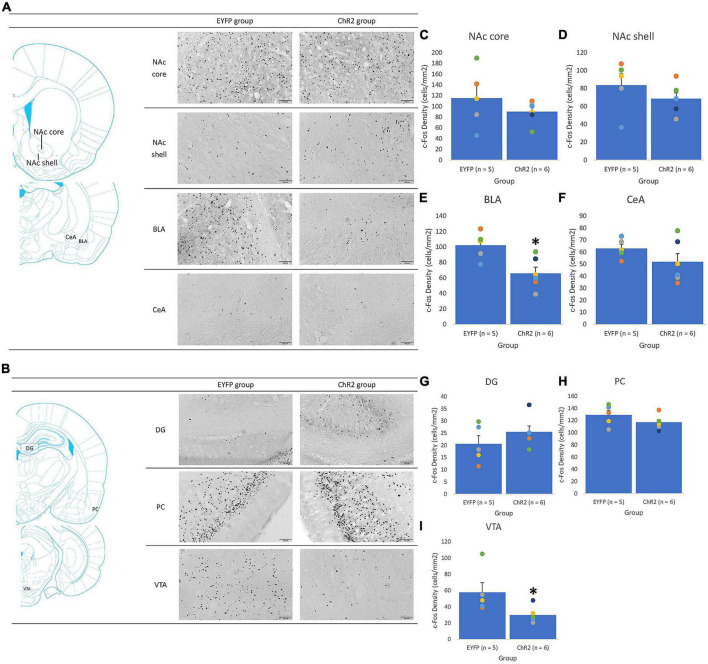
The PrL photostimulation was given in conditioning. An illustrated brain atlas showing **(A)** the nucleus accumbens core (NAc core), nucleus accumbens shell (NAc shell), basolateral amygdala (BLA), and central amygdala (CeA); **(B)** the dentate gyrus (DG), piriform cortex (PCl), and ventral tegmental area (VTA); c-Fos density in the **(C)** NAc, **(D)** NAc shell, **(E)** BLA, **(F)** CeA, **(G)** DG, **(H)** PC, and **(I)** VTA for the EYFP (*n* = 5) and ChR2 (*n* = 6) groups after PrL optical stimulation and morphine-induced CTA in extinction. *p < 0.05 compared with the EYFP group.

**FIGURE 8:**
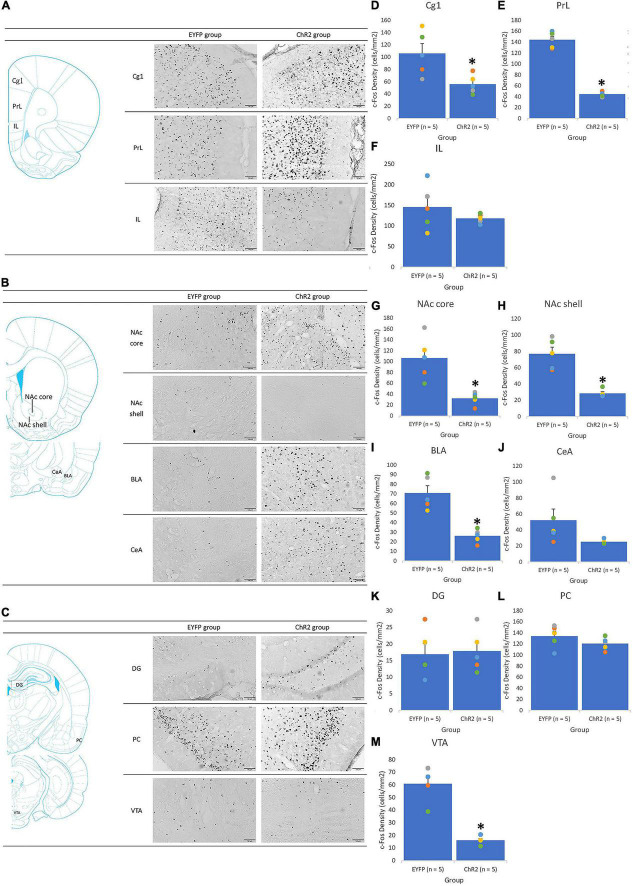
The IL photostimulation was given in conditioning. An illustrated brain atlas showing **(A)** the cingulate cortex 1 (Cg1), prelimbic cortex (PrL), and infralimbic cortex (IL); **(B)** the nucleus accumbens core (NAc core), nucleus accumbens shell (NAc shell), basolateral amygdala (BLA), and central amygdala (CeA); **(C)** the dentate gyrus (DG), piriform cortex (PCl), and ventral tegmental area (VTA); c-Fos density in the **(D)** Cg1, **(E)** PrL, **(F)** IL, **(G)** NAc core, **(H)** NAc shell, **(I)** BLA, **(J)** CeA, **(K)** DG, **(L)** PC, and **(M)** VTA for the EYFP (*n* = 5) and ChR2 (*n* = 5) groups after IL optical stimulation and morphine-induced CTA in conditioning tests. *p < 0.05 compared with the EYFP group.

After ChR2 photostimulation was performed on the Cg1, PrL, and IL, it affected morphine-induced aversive saccharin solution suppression during conditioning. Later, selective neural substrates were labeled by immunohistochemical staining of the c-Fos protein to indicate neural activity associated with the behavioral test (Figures 6–8).

Immunohistochemical staining with c-Fos protein was performed in selective brain areas to determine which neural substrates were involved in c-Fos expression under optical stimulation of the Cg1, PrL, and IL after morphine-induced conditioned suppression of saccharin solution intake during conditioning and extinction. This was based on behavioral data that indicated whether saccharin solution consumption was altered under optical stimulation of the Cg1, PrL, and IL in conditioning and under optical stimulation of the PrL in extinction. Therefore, only c-Fos data in the Cg1, PrL, and IL photostimulation conditioning experiments and the PrL photostimulation in extinction were evaluated by one-way ANOVA. Cg1 and IL optical stimulation did not affect morphine-induced saccharin solution consumption in extinction. Therefore, the c-Fos data of the Cg1 and IL following optical stimulation in extinction were not analyzed and are not presented in this study.

Optogenetic photostimulation in the target areas activated the involvement of neural substrates in aversive, morphine-induced saccharin solution consumption. In Cg1 photostimulation and conditioning tests, the ChR2 group exhibited a significant decrease in c-Fos density in the PrL [*F*_(1, 8)_ = 6.34, *p* < 0.05; Figure 6D], IL [*F*_(1, 8)_ = 10.07, *p* < 0.05; Figure 6E], NAc shell [*F*_(1, 8)_ = 9.78, *p* < 0.05; Figure 6G], and CeA [*F*_(1, 8)_ = 10.40, *p* < 0.05; Figure 6I]; however, a significant increase in c-Fos density was observed in the BLA [*F*_(1, 8)_ = 23.06, *p* < 0.05; Figure 6H] following one-way ANOVA analysis. Non-significant differences in c-Fos density between the ChR2 and EYFP groups in the Cg1 [*F*_(1, 8)_ = 1.32, *p* > 0.05; Figure 6C] and NAc core [*F*_(1, 8)_ = 3.07, *p* > 0.05; Figure 6F] were found using one-way ANOVA analysis. Therefore, following Chr2 photostimulation of Cg1, c-Fos expression was downregulated in the PrL, IL, NAc shell, and CeA and upregulated in the BLA. This suggests that the PrL, IL, NAc shell, CeA, and BLA are involved in the mechanism by which Cg1 photostimulation enhances conditioned, morphine-induced aversive saccharin solution consumption. However, the BLA played an opposite role to the other neural substrates in this regard.

In the PrL photostimulation and conditioning tests, c-Fos data revealed significant decreases in c-Fos density in the BLA [*F*_(1, 9)_ = 9.75, *p* < 0.05; Figure 7E] and VTA [*F*_(1, 9)_ = 5.53, *p* < 0.05; Figure 7I] for the ChR2 group compared with the EYFP group; moreover, non-significant differences were observed in the NAc core [*F*_(1, 9)_ = 1.11, *p* > 0.05; Figure 7C], NAc shell [*F*_(1, 9)_ = 1.23, *p* > 0.05; Figure 7D], CeA [*F*_(1, 9)_ = 1.73, *p* > 0.05; Figure 7F], DG [*F*_(1, 9)_ = 1.43, *p* > 0.05; Figure 7G], and PC [*F*_(1, 9)_ = 1.93, *p* > 0.05; Figure 7H] between the EYFP and ChR2 groups. Therefore, ChR2 photostimulation of PrL facilitated morphine-induced aversive saccharin solution consumption in conditioning tests. Moreover, c-Fos expression was upregulated in the BLA and downregulated in the VTA.

In conditioning tests, IL photostimulation of the ChR2 group elicited a significant decrease in c-Fos expression in the Cg1 [*F*_(1, 8)_ = 8.27, *p* < 0.05; Figure 8D], PrL [*F*_(1, 8)_ = 217.01, *p* < 0.05; Figure 8E], NAc core [*F*_(1, 8)_ = 16.21, *p* < 0.05; Figure 8G], NAc shell [*F*_(1, 8)_ = 32.29, *p* < 0.05; Figure 8H], BLA [*F*_(1, 8)_ = 28.97, *p* < 0.05; Figure 8I], and VTA [*F*_(1, 8)_ = 54.41, *p* < 0.05; Figure 8M] compared with the EYFP group. However, no significant differences were observed between the EYFP and ChR2 groups in the IL [*F*_(1, 8)_ = 1.23, *p* > 0.05; Figure 8F], CeA [*F*_(1, 8)_ = 3.64, *p* > 0.05; Figure 8J], DG [*F*_(1, 8)_ = 0.05, *p* > 0.05; Figure 8K], and PC [*F*_(1, 8)_ = 1.63, *p* > 0.05; Figure 8L]. In conclusion, ChR2 photostimulation of the IL increased morphine-induced, aversive saccharin solution consumption; moreover, c-Fos density was downregulated in the Cg1, PrL, NAc core, NAc shell, BLA, and VTA.

**FIGURE 9:**
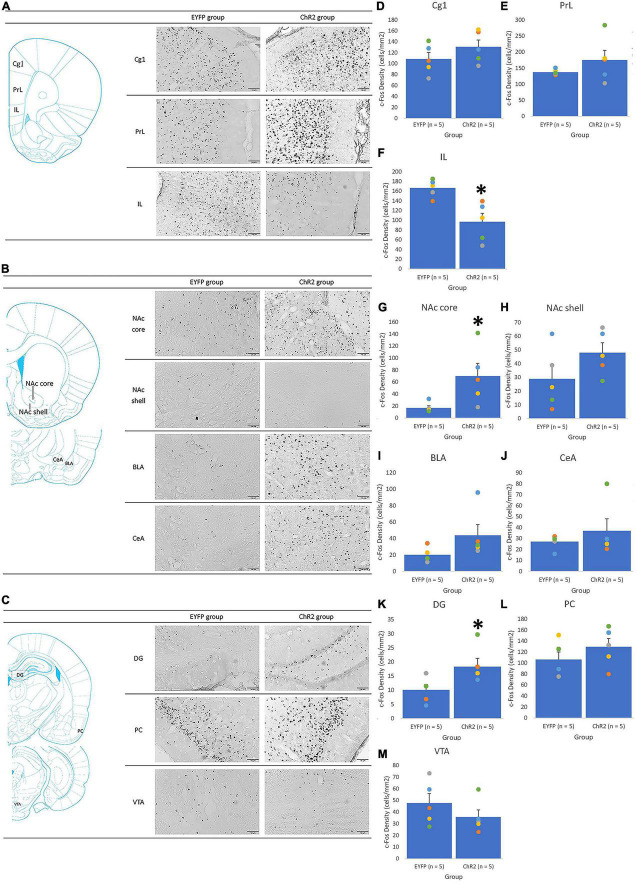
The PrL photostimulation was given in extinction. An illustrated brain atlas showing **(A)** the cingulate cortex 1 (Cg1), prelimbic cortex (PrL), and infralimbic cortex (IL); **(B)** the nucleus accumbens core (NAc core), nucleus accumbens shell (NAc shell), basolateral amygdala (BLA), and central amygdala (CeA); **(C)** the dentate gyrus (DG), piriform cortex (PCl), and ventral tegmental area (VTA). c-Fos density in the **(D)** Cg1, **(E)** PrL, **(F)** IL, **(G)** NAc core, **(H)** NAc shell, **(I)** BLA, **(J)** CeA, **(K)** DG, **(L)** PC, and **(M)** VTA for the EYFP (*n* = 5) and ChR2 (*n* = 5) groups after PrL optical stimulation and morphine-induced CTA in extinction. *p < 0.05 compared with the EYFP group.

After ChR2 photostimulation was performed on the Cg1, PrL, and IL, the extinction effect was impacted following morphine-induced aversive saccharin solution suppression. However, only the PrL decreased the extinction effect; however, the Cg1 and IL photostimulation did not affect the extinction effect at the behavioral level. Thus, the c-Fos data are only shown for ChR2 photostimulation of the PrL and for the extinction phase (Figure 9).

In the extinction tests, only PrL photostimulation elicited the extinction effect of aversive saccharin solution consumption induced by morphine. Cg1 and IL photostimulation did not affect this. Accordingly, the results only include the involvement of PrL photostimulation. For example, a significant decrease in c-Fos density was observed in the IL for the ChR2 group [*F*_(1, 8)_ = 12.64, *p* < 0.05; Figure 9F] and a significant increase in c-Fos density was observed in the NAc core [*F*_(1, 8)_ = 6.12, *p* < 0.05; Figure 9G] and DG [*F*_(1, 8)_ = 5.27, *p* = 0.05; Figure 9K] for the ChR2 group compared with the EYFP group; however, no significant difference in c-Fos density was observed between the EYFP and ChR2 groups in the Cg1 [*F*_(1, 8)_ = 1.51, *p* > 0.05; Figure 9C], PrL [*F*_(1, 8)_ = 1.42, *p* > 0.05; Figure 9E], NAc shell [*F*_(1, 8)_ = 2.49, *p* > 0.05; Figure 9H], BLA [*F*_(1, 8)_ = 2.99, *p* > 0.05; Figure 9I], CeA [*F*_1,8_ = 0.80, *p* > 0.05; Figure 9J], PC [*F*_(1, 8)_ = 1.24, *p* > 0.05; Figure 9L], or VTA [*F*_(1, 8)_ = 1.30, *p* > 0.05; Figure 9M]. Therefore, ChR2 photostimulation of the PrL in extinction tests decreased the extinction effect of morphine-induced aversive saccharin solution consumption; moreover, c-Fos expression was downregulated in the IL and upregulated in the NAc core and DG.

## 4. Discussion

The study modified the previous study that used the rewarding valence of saccharin solution intake and morphine-induced aversive valence of saccharin solution intake in conditioning (23); moreover, the neutral states of saccharin solution was performed by the extinction procedure. Optogenetic stimulation of the Cg1, PrL, and IL modulates the rewarding, aversive, and neutral valences of saccharin solution consumption associated with morphine administration in conditioning and extinction. In this study, optogenetic stimulation of Cg1 increased the rewarding valence of saccharin solution consumption and the aversive valence of saccharin solution consumption induced by morphine in conditioning tests. Optogenetic stimulation of the PrL decreased the rewarding valence of saccharin solution consumption, increased the aversive valence of saccharin solution consumption induced by morphine conditioning, and decreased the neutral valence of saccharin solution consumption in morphine-induced extinction. IL optogenetic stimulation increased the aversive valence of saccharin solution consumption induced by morphine in conditioning (Table 3). Importantly, it should be noted that this valence change (from the reward and aversion to neutral status) of the stimulus was temporary but not permanent alternation in saccharin solution consumption during light-on relative to the light-off period during the session.

**TABLE 3:** Changes in saccharin solution consumption by valence, from rewarding/aversive to neutral, under optogenetic ChR2 excitatory stimulation in the Cg1, PrL, and IL.

	Rewarding valence → Aversive valence → Neutral valence		
Cg1	↑	↑	—
PrL	↓	↑	↓
IL	—	↑	—

—, non-significant differences; ↓, decrease; ↑, increase; Cg1, cingulate cortex 1; PrL, prelimbic cortex; IL, infralimbic cortex.

Regarding optogenetic Cg1 stimulation in morphine-induced, aversive saccharin solution consumption suppression in conditioning tests, increased c-Fos expression was observed in the BLA, and decreased c-Fos expression was observed the PrL, IL, NA shell, and CeA. Following PrL optogenetic stimulation, increased c-Fos expression was observed in the BLA and VTA in conditioning tests. In extinction tests, PrL optogenetic stimulation increased c-Fos expression in the NAc core and decreased c-Fos expression in the IL and DG. IL optogenetic stimulation decreased c-Fos expression in the Cg1, PrL, NAc core, NAc shell, BLA, and VTA in conditioning tests. Because the Cg1 and IL photostimulation did not affect the extinction effect, the data of c-Fos expression were excluded. Thus, it was shown no application in Table 4. In conditioning, the Cg1 photostimulation inhibited c-Fos expression in the PrL and IL; the IL photostimulation also inhibited c-Fos expression in the Cg1 and PrL. However, the PrL photostimulation did not interact with c-Fos expression in the Cg1 and IL (Table 4). Therefore, the subareas of the mPFC might interact with each other to modulate the aversive saccharin solution consumption suppression by morphine.

**TABLE 4:** c-Fos expression after morphine-induced aversive saccharin solution consumption suppression in conditioning and extinction following optogenetic ChR2 excitatory stimulation in the Cg1, PrL, and IL.

		Conditioning			Extinction	
c-Fos expression	—	↑	↓	—	↑	↓
Cg1	Cg1, NAc core, DG, VTA	BLA	PrL, IL, NAc shell, CeA	N/A	N/A	N/A
PrL	Cg1, PrL, IL, NAc core, NAc shell, CeA, DG	—	BLA, VTA	Cg1, PrL, NAc shell, BLA, CeA, VTA	NAc core	IL, DG
IL	IL, CeA, DG	—	Cg1, PrL, NAc core, NAc shell, BLA, VTA	N/A	N/A	N/A

N/A, not applicable; —, non-significant differences; ↓, decrease; ↑, increase; Cg1, cingulate cortex 1; PrL, prelimbic cortex; IL, infralimbic cortex; BLA, basolateral amygdala; NAc core, nucleus accumbens core; NAc shell, nucleus accumbens shell; CeA, central amygdala; VTA, ventral tegmental area; DG, dentate gyrus.

### 4.1. Comparison of previous findings with the present data associated with subareas of the mPFC modulating the rewarding, aversive, and neutral valences

Many neural substrates have previously been shown to regulate the rewarding and aversive valences of stimuli through different mechanisms (27–30). The major focus of previous studies has been the mesolimbic dopamine system governing reward and aversion properties (17, 28, 29, 31–35). For example, a previous review suggested that the mesolimbic dopamine system contributed to the valence of rewarding, aversive, and altering stimuli (28). Another review reported that optogenetic stimulation of GABAergic and glutamatergic circuits in the VTA and limbic system triggered reinforcement and aversion in motivated behaviors (29). The cannabinoid neurons of the VTA were shown to contribute to aversion and rewarding attenuation, but not to reinforcement or rewarding enhancement (31). Electrophysiological recording in the NAc revealed an increased response to the rewarding sucrose stimulus. However, the NAc neurons that were responsive to sucrose solution intake exhibited a significant increase in firing rate after sucrose was conditioned with aversive lithium chloride, indicating that the NAc responded to both the aversive stimulus and the rewarding stimulus (35). Microinjections of the metabotropic Group II receptor (mgly2/3) antagonist LY341495 in the NAc shell suppressed the positive, affinitive reaction and increased aversive behaviors, indicating that metabotropic glutamate receptor antagonism in the NAc shell induced a valence shift from reward to aversion (34). The midbrain dopamine system, from the VTA to the NAc, has dopamine subpopulations that are heterogeneous in both structure and function, and has been found to be involved in reward processing and aversive events (32). The laterodorsal tegmental neurons and the lateral habenula project to the VTA and control reward and aversion, respectively; that is, the laterodorsal tegmentum projects to the VTA and conveys information to the NAc shell to regulate reward (33). The lateral habenula connects to the VTA and synapses with the mPFC to control aversion (33). Therefore, the mesolimbic dopamine system from the VTA to the NAc projection governs the valence of both reward and aversion.

In contrast to existing studies on the role of mesolimbic dopamine system in reward and aversion, less evidence exists regarding the contribution of the amygdala to the valence of reward and aversion (27, 30). For example, in a previous study, distinct neurons in the BLA increased firing rates to aversive and rewarding conditioning, indicating that those subsets of BLA neurons regulate the specific valence of stimuli (30). The amygdala and orbitofrontal cortex exhibited a dynamic relationship to regulate the reward and aversion valences resulting in approach and avoidance behavior (27).

The issue of how the mPFC modulates the valence of reward and aversion remains diverse. For example, inhibition of the PrL has been shown to decrease active but not inhibitory avoidance behaviors in a discriminative task, while inactivation of the IL impaired active and inhibitory avoidance behaviors in this regard. Moreover, PrL and IL inactivation disrupted inhibitory behaviors but not active reward-seeking behaviors in a cued rewarding go/no-go task, indicating that the PrL and IL have different roles in regulating reward and aversion (19). Glial modulation of the mPFC has been shown to directly regulate extracellular glutamate release for the rewarding valence in the intracranial self-administration task and aversive valence in aversive stimulation in the immobilization stress task (36). A previous study demonstrated that CB1 transmission in the PrL altered the morphine reward valence to an aversive valence; moreover, CB1 transmission antagonism increased the rewarding valence in CPP conditioning induced by morphine, indicating that the activation or inhibition of CB1 transmission in the PrL bidirectionally modulated opiate-induced rewarding and aversive valences (20). Therefore, the mPFC subareas appear to play a disparate role in modulating the valence of reward and aversion. The present data partially support previous findings suggesting that the mPFC integrates valence and action (17) and that subareas of the mPFC (e.g., Cg1, PrL, and IL) play disparate roles in the valence of reward and aversion (13).

Additionally, the mesolimbic dopamine system (including the NAc, VTA, and amygdala) and subareas of the mPFC (e.g., Cg1, PrL, and IL) contribute to the valence of reward and aversion. In this study, mPFC subareas exhibited distinct functions regulating the valence of reward and aversion to neutral properties.

### 4.2. c-Fos data on the modulation of mPFC subareas: Comparing the present findings and existing data

The present study revealed that excitation of mPFC subareas (e.g., Cg1, PrL, and IL) with optogenetic ChR2 approaches partially inhibited the neuronal activity of subareas of the mPFC, NAc, amygdala, and VTA *via* labeling with c-Fos proteins. For example, Cg1 optogenetic stimulation decreased c-Fos expression in the PrL and IL of the mPFC, NAc shell, and CeA of the amygdala, PrL optogenetic stimulation decreased c-Fos expression in the BLA and VTA, and IL optogenetic stimulation suppressed c-Fos expression in the Cg1 and PrL of the mPFC, NAc core, NAc shell, BLA, and VTA in aversive saccharin solution suppression induced by morphine in conditioning tests. Therefore, the present data suggest that the glutamatergic neurons of the mPFC convey top-down control and information to the mesolimbic dopamine system, including the NAc, amygdala, and VTA. The present findings are in line with previous observations that the mPFC interacts highly with subcortical brain areas, such as the thalamus, amygdala, striatum, and hippocampus, and that it exerts top-down executive control of various cognitive functions, including attention, inhibitory control behaviors, habit formation, and working, spatial, and long-term memory (37). Moreover, the mPFC is suggested to exert top-down control of the processing of rewarding and aversive stimuli (38). Therefore, it can be concluded that mPFC subareas modulate the valence of reward and aversion. However, how the mPFC subareas project to subcortical regions, such as the amygdala, NAc, and VTA, to regulate rewarding and aversive valence should be examined in further studies.

The present data indicate that optogenetic PrL stimulation decreased c-Fos expression in the BLA compared with the EYFP group. This result was identical to previous studies reporting that excitation of the PrL with low-concentration NMDA injections reduced the aversive saccharin solution consumption induced by morphine administration. Moreover, c-Fos expression was lower in the BLA after morphine conditioning, indicating that the interaction between the PrL and BLA plays a balancing role in morphine-induced aversive saccharin solution suppression in conditioning (39). Furthermore, other research has demonstrated that the connections of the PrL and BLA are involved in reward processing and aversively conditioned learning (40, 41). For example, in a previous study, hypofunction of NMDA receptors in the PrL facilitated sensitivity in the valence of reward through BLA dopaminergic transmission in opiate-induced CPP learning (40). The projection of the PrL to the VLA has also mediated the retrieval effect of morphine withdrawal memory in aversively conditioned place aversion learning (41). Therefore, connection of the PrL neurons to the BLA likely mediates the rewarding and aversive valence in conditioned learning.

The balancing role of the PrL also interacts with the VTA in aversive saccharin solution consumption induced by morphine in conditioning. Moreover, the PrL plays a balancing role with the IL and the DG of the hippocampus in morphine extinction. How the PrL connects with the VTA, IL, and DG of the hippocampus to regulate the valence of rewarding, aversive, and neutral properties remains unclear. This crucial issue should be investigated in further studies.

### 4.3. Experimental limitations and emerged issues

This study suggests that the subareas of the mPFC (e.g., Cg1, PrL, and IL) were involved in the valence of the rewarding, aversive, and neutral stimulus in saccharin solution consumption. For example, ChR2 photostimulation of Cg1 and PrL, respectively, increased and decreased the rewarding valence of the saccharin solution intake. Optogenetic photostimulation of the Cg1, PrL, and IL interfered with morphine-induced saccharin solution consumption, indicating that the mPFC’s subareas downregulated the aversive valence. Furthermore, morphine-induced saccharin solution extinction was decreased under PrL photostimulation, but not under the Cg1 and IL of the mPFC. However, recent studies have shown the mPFC to modulate thirst or hunger state to drive motivational behaviors, and this potential confounding variable might affect saccharine consumption (42, 43). Therefore, whether this confounding variable would influence the present results should be of concern in future studies.

Additionally, in this study, there emerged the issue of whether longer duration of ChR2 photostimulation would destroy neurons in the target brain site. The optical parameters of the present study used 15 ms, 20 Hz pulses for 3 min for ChR2 photostimulation. The experimental procedure of the saccharin solution consumption requires a longer drinking time, and thus, we used a time of 3 min to measure saccharin solution consumption. Although the procedure of ChR2 photostimulation was based on the previous study (24), there remains the opportunity to destroy the neurons of the target brain areas with heating. Therefore, future studies should address this possibility.

In light of the c-Fos data, we interestingly found that the inhibition of the Cg1, PrL, and IL *via* ChR2 photostimulation activated the neural activity of numerous brain areas, including the subareas of the mPFC, amygdala, NAc, and VTA. These findings indicate that the inhibition of the Cg1, PrL, or IL would change the neural network among the mPFC, amygdala, NAc, and VTA. Conversely, the enhancement of the subareas of the mPFC, including Cg1, PrL, and IL, activated fewer brain areas, such as the BLA and NAc core. Therefore, the Cg1, PrL, and IL likely contributed inhibitively to modulate the neural network for the mPFC, amygdala, NAc, and VTA. This topic should be examined in further studies.

In Experiment 2, the results showed that the total intake volume of saccharin solution was increased from light-off to light-on for the EYFP group in photostimulation of the Cg1, PrL, and IL in conditioning or extinction (Figures 6A–F). The results might be because of taste neophobia (44–46). For example, the previous behavioral study demonstrated that the novel taste solution intake would be reduced in the initial phase; however, when the novel taste was familiar, the intake volume of taste solution appeared to increase, indicating it is the taste neophobia (45). It explains why the taste solution intake is enhanced. In summary, changing the total intake volume of saccharin solution in the EYFP group from decreases to increases might be due to taste neophobia. This issue of why taste neophobia affects the saccharin solution intake in the early stage should be scrutinized further.

The c-Fos data showed that c-Fos density in the specific brain areas under photostimulation of the Cg1, PrL, and IL in conditioning or extinction; however, the present study did not design a no-stimulation control group. The experiment used EYFP virus infection and photostimulation, which was a control group. However, it might produce confounding effects to interfere with the c-Fos data and its results. The photostimulation in the specific brain areas might destroy the neurons within the specific brain areas, thereby reducing the c-Fos expression. Therefore, the no-stimulation control group should be considered in further studies.

On the other hand, c-Fos expression was examined on the final day after behavioral testing; however, this day was not given any photostimulation. Based on the experimental procedure, it is reasonable that the c-Fos data might be combined with the effects of behavioral tests and photostimulation. Therefore, the issue of dissociating the effects from the behavioral tests and photostimulation was crucial in further research.

The present study examined whether photostimulation in the Cg1, PrL, and IL of the mPFC modulated the altered valence from reward with the innate saccharin solution consumption and aversion induced by morphine conditioning to neutral status through conditioning extinction process for the saccharin solution consumption. The present data cannot be ruled out the effect of conditioning or memory retention in our animal model because we use the conditioning procedure and extinction process to alter the valence of the saccharin solution from the original reward, conditioned aversion, and finally, extinction process due to neutral status. Therefore, an issue emerged that whether the experimental procedure can be designed to fully remove the effect of conditioning or memory retention from the valence of stimulus remains to be scrutinized in further studies.

Another issue should be concerned. The present study used photostimulation into the Cg1, PrL, and IL to modulate the changing valence of the stimulus from reward and aversion to neutral status. Furthermore, Experiment 2 examined the neural substrates in the aversive and neutral valence of the saccharin solution using immunohistochemical staining with c-Fos expression after conditioning or the extinction process. However, the present study did not test the same experiment without optical stimulation or inhibition and examined the normal function circuits. This issue should be examined in further research.

## 5. Conclusion

How the valence of the stimulus changes from rewarding and aversive to neutral statuses may crucial for ameliorating drug addiction. We found that the subareas of the medial prefrontal cortex (e.g., Cg1, PrL, and IL) played different roles in modulating the valence of stimulus. Understanding how to alter the rewarding property of abused drugs to aversive from neutral properties is critical. Altering stimulus valences may reduce drug relapse and drug-seeking in addictive behaviors. Here, optogenetic Cg1, PrL, and IL stimulation *via* glutamatergic neurons modulated alterations in stimulus valences and could aid in the development of novel treatments for the amelioration of addictive symptoms.

## Data availability statement

The raw data supporting the conclusions of this article will be made available by the authors, without undue reservation.

## Ethics statement

The animal study was reviewed and approved by the Fo Guang University Institutional Animal Care and Use Committee.

## Author contributions

YY and CO: methodology, investigation, and project administration. AT: methodology, validation, and formal analysis. C-NC and FC: project administration. BS: writing—review and editing, investigation, supervision, and funding acquisition. AH: conceptualization, methodology, formal analysis, writing—original draft, writing—review and editing, supervision, and funding acquisition. All authors contributed to the article and approved the submitted version.

## Supplementary material

Supplementary Figure 1 Optogenetic verification of Cg1, PrL, and IL photostimulation. **(A)** Left: the brain atlas depicting the locations of optical stimulation in the Cg1, PrL, and IL. Right: AAV virus infection with the CaMKII promotor in fluorescent green, c-Fos expression in red, and merged CaMKII and c-Fos expression in the Cg1, PrL, and IL. White boxes indicate c-Fos expression neurons with photostimulation in the Cg1, PrL, and IL. c-Fos expression neurons are indicated with arrowheads in the upper square images. **(B)** The location of the buried optical fibers. Blue spots indicate the Cg1 optical stimulation location, orange spots indicate the PrL optical stimulation location, and green spots indicate the IL optical stimulation location. Cg1, cingulate cortex 1; PrL, prelimbic cortex; IL, infralimbic cortex.

Supplementary Figure 2 Optogenetic verification of Cg1, PrL, and IL photostimulation. **(A)** Left: AAV virus infection with the CaMKII promotor is shown in fluorescent green, c-Fos expression is shown in red; merged CaMKII and c-Fos expression is shown in the Cg1, PrL, and IL. Middle: brain atlas depicting locations of optical stimulation in the Cg1, PrL, and IL. **(B)** c-Fos expression (%) following optical stimulation of the Cg1, PrL, and IL between the EYFP and ChR2 groups. **(C)** The location of the buried optical fibers in the EYFP and ChR2 groups during the conditioning and extinction phases. Blue spots depict Cg1 optical stimulation location, orange spots depict PrL optical stimulation location, and green spots depict IL optical stimulation location in the EYFP group during conditioning and extinction. Gray spots depict Cg1 optical stimulation location, brown spots depict PrL optical stimulation location, and dark green spots depict IL optical stimulation location in the ChR2 group during conditioning and extinction. Cg1, cingulate cortex 1; PrL, prelimbic cortex; IL, infralimbic cortex.
