# Supplementary figures and images for: Optogenetic stimulation in the medial prefrontal cortex modulates stimulus valence from rewarding and aversive to neutral states
Source: Front Psychiatry. 2023 Apr 11;14:1119803. doi: 10.3389/fpsyt.2023.1119803 (PMC10126430; doi:10.3389/fpsyt.2023.1119803)

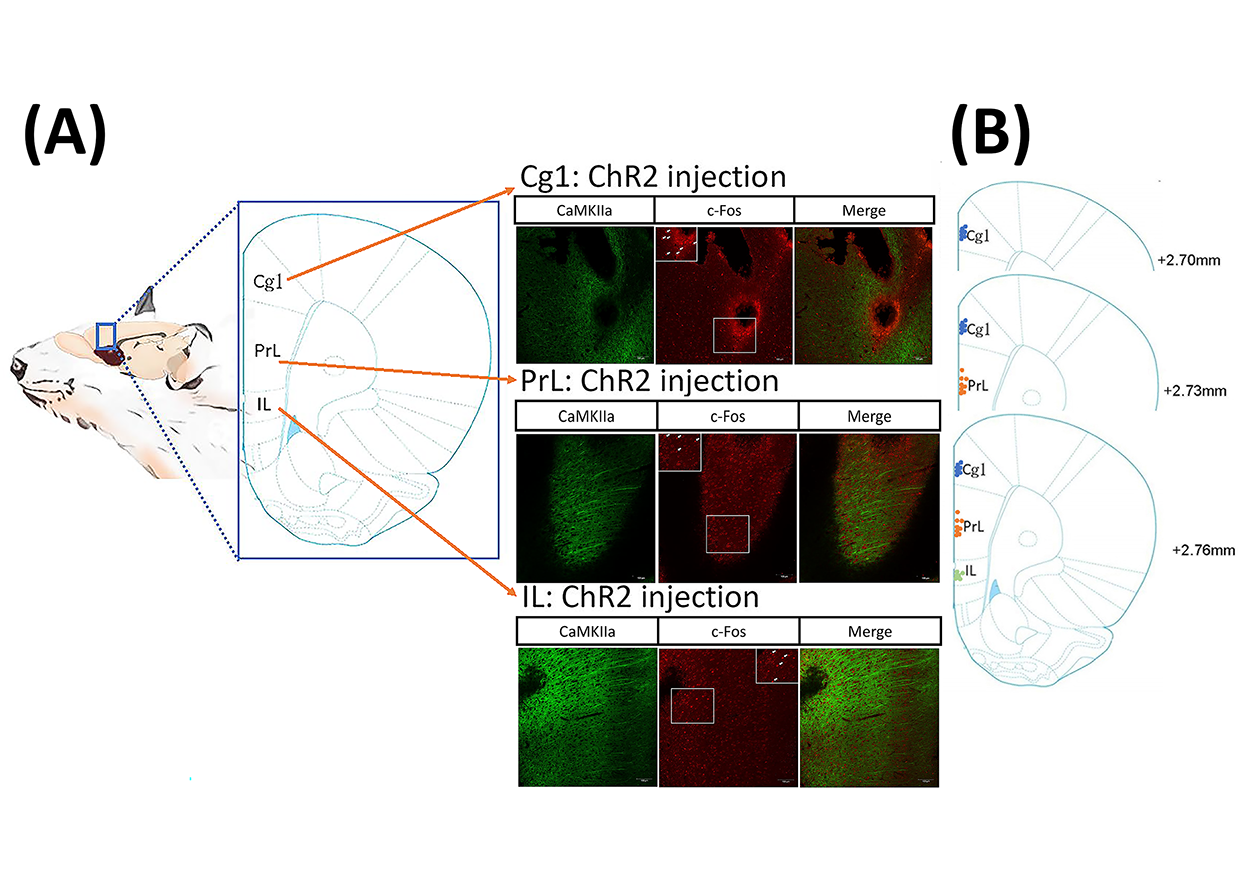

Supplement: Supplementary Figure 1 — Optogenetic verification of Cg1, PrL, and IL photostimulation. (A) Left: the brain atlas depicting the locations of optical stimulation in the Cg1, PrL, and IL. Right: AAV virus infection with the CaMKII promotor in fluorescent green, c-Fos expression in red, and merged CaMKII and c-Fos expression in the Cg1, PrL, and IL. White boxes indicate c-Fos expression neurons with photostimulation in the Cg1, PrL, and IL. c-Fos expression neurons are indicated with arrowheads in the upper square images. (B) The location of the buried optical fibers. Blue spots indicate the Cg1 optical stimulation location, orange spots indicate the PrL optical stimulation location, and green spots indicate the IL optical stimulation location. Cg1, cingulate cortex 1; PrL, prelimbic cortex; IL, infralimbic cortex. [file Image_1.TIF]

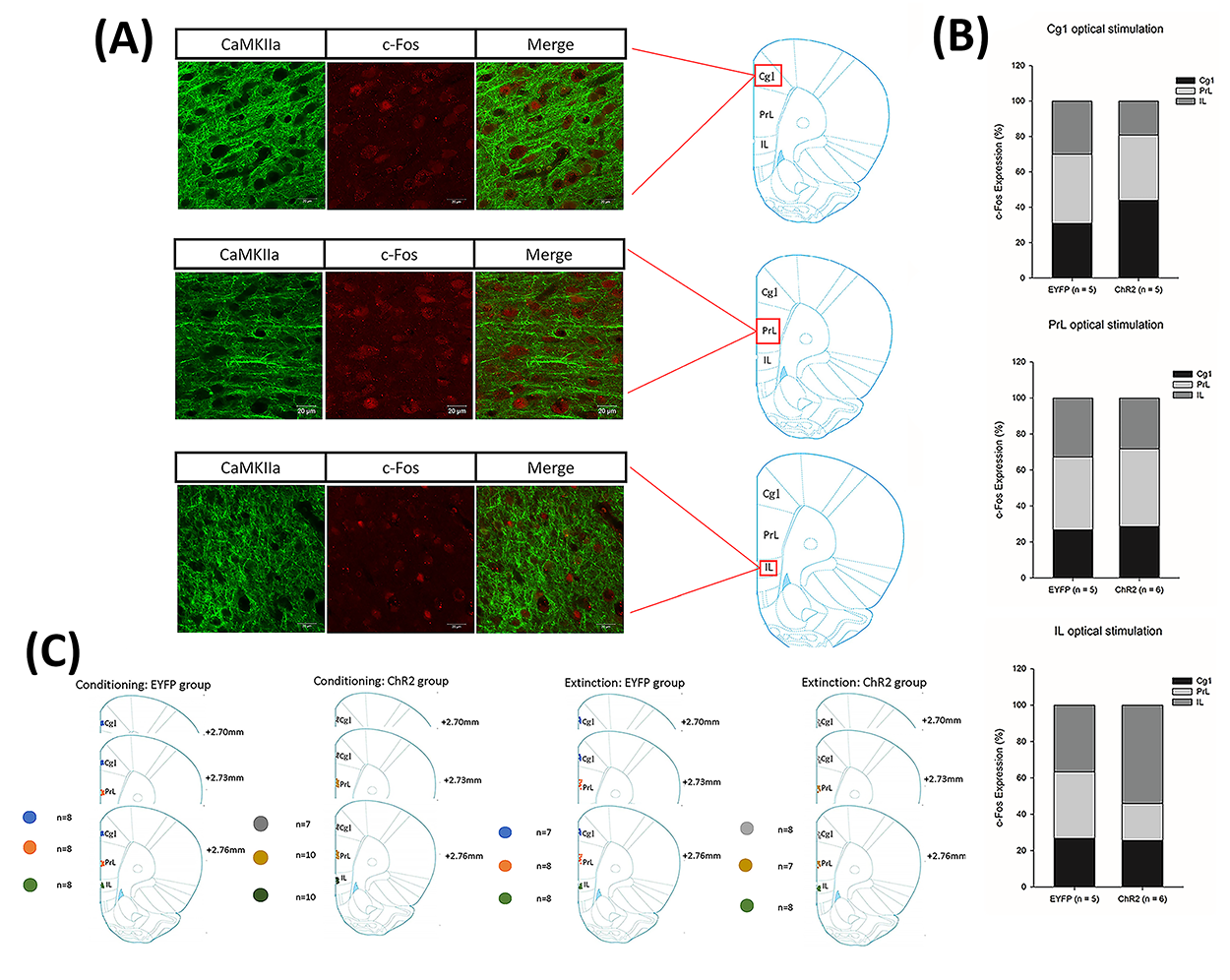

Supplement: Supplementary Figure 2 — Optogenetic verification of Cg1, PrL, and IL photostimulation. (A) Left: AAV virus infection with the CaMKII promotor is shown in fluorescent green, c-Fos expression is shown in red; merged CaMKII and c-Fos expression is shown in the Cg1, PrL, and IL. Middle: brain atlas depicting locations of optical stimulation in the Cg1, PrL, and IL. (B) c-Fos expression (%) following optical stimulation of the Cg1, PrL, and IL between the EYFP and ChR2 groups. (C) The location of the buried optical fibers in the EYFP and ChR2 groups during the conditioning and extinction phases. Blue spots depict Cg1 optical stimulation location, orange spots depict PrL optical stimulation location, and green spots depict IL optical stimulation location in the EYFP group during conditioning and extinction. Gray spots depict Cg1 optical stimulation location, brown spots depict PrL optical stimulation location, and dark green spots depict IL optical stimulation location in the ChR2 group during conditioning and extinction. Cg1, cingulate cortex 1; PrL, prelimbic cortex; IL, infralimbic cortex. [file Image_2.TIF]
